# Mini-review: The neurobiology of treating substance use disorders with classical psychedelics

**DOI:** 10.3389/fnins.2023.1156319

**Published:** 2023-04-17

**Authors:** Marvin M. Urban, Moritz R. Stingl, Marcus W. Meinhardt

**Affiliations:** ^1^Institute of Psychopharmacology, Central Institute of Mental Health, Medical Faculty Mannheim, University of Heidelberg, Mannheim, Germany; ^2^Interdisciplinary Center for Neurosciences, University of Heidelberg, Heidelberg, Germany; ^3^Department of Molecular and Cellular Biology, Harvard University, Cambridge, MA, United States; ^4^Department of Molecular Neuroimaging, Central Institute of Mental Health, Medical Faculty Mannheim, University of Heidelberg, Mannheim, Germany

**Keywords:** psychedelics, addiction, psilocybin, hallucinogen, substance, dependency, plasticity, therapy

## Abstract

The potential of psychedelics to persistently treat substance use disorders is known since the 1960s. However, the biological mechanisms responsible for their therapeutic effects have not yet been fully elucidated. While it is known that serotonergic hallucinogens induce changes in gene expression and neuroplasticity, particularly in prefrontal regions, theories on how specifically this counteracts the alterations that occur in neuronal circuitry throughout the course of addiction are largely unknown. This narrative mini-review endeavors to synthesize well-established knowledge from addiction research with findings and theories regarding the neurobiological effects of psychedelics to give an overview of the potential mechanisms that underlie the treatment of substance use disorders with classical hallucinogenic compounds and point out gaps in the current understanding.

## Introduction

In 2019, substance use disorders (SUDs) affected an estimated 2.2% of global population and continue to impose a substantial economic burden on society (Peacock et al., 2018; Castaldelli-Maia and Bhugra, 2022). Drug addiction is commonly described as a behavioral disorder featuring cycles of abuse, abstinence, and relapse, as well as negative affective states (Kalivas et al., 2009; Wolf, 2016; Koob, 2022). SUDs result from repeated exposure of a vulnerable brain to reinforcing drugs. This induces various transcriptional and epigenetic changes, altering neural pathways that mediate reward assignment, motivation, and executive control (Nestler, 2001; Lüscher and Malenka, 2011; Ruffle, 2014). The resulting behavioral changes are very stable, potentially persisting over the lifetime of an individual and leading to relapses even after years of abstinence (Spanagel, 2009).

Current pharmacotherapies for SUDs include agonist replacement, reduction of withdrawal symptoms, and inhibition of the rewarding properties of addictive drugs (Volpicelli et al., 1995; Warner and Shoaib, 2005; Lobmaier et al., 2010). However, so far, these approaches are limited to certain drugs or classes of drugs because they target receptors or enzymes specific to particular substances, instead of the underlying pathway modifications shared by all SUDs (Kreek et al., 2002; Nichols et al., 2017).

Recently, serotonergic hallucinogens (here also referred to as psychedelics), such as psilocybin, lysergic acid diethylamide (LSD), or *N,N*-dimethyltryptamine (DMT), have shown great promise in reducing symptoms of substance addictions and mood disorders, as indicated by a vast body of research and reviews (Dos Santos et al., 2018; Romeo et al., 2021; Bogenschutz et al., 2022; Calleja-Conde et al., 2022; Jones et al., 2022; Mendes et al., 2022; van der Meer et al., 2023). Hypotheses explaining the therapeutic efficacy of these drugs cover psychosocial models, large-scale effects on neural activity and network connectivity, neuroinflammatory mechanisms, as well as molecular/pharmacological actions and are, thus, as multilayered as the phenomenon of addiction (Tófoli and de Araujo, 2016; Koslowski et al., 2022; Teixeira et al., 2022; van Elk and Yaden, 2022). Among these explanations, the induction of structural neuroplasticity is often pointed out as the key mechanism (Mertens and Preller, 2021; Nutt et al., 2023). This mini-review focuses on neuroplastic changes in addiction circuitry and how they potentially lead to alleviation of symptom load.

## The addicted brain

As addiction develops, successive adaptations in distinct but interconnected neurocircuits take place while compulsivity of drug intake gradually increases. The affected circuits comprise reward-related pathways, prefrontal control networks, and stress systems (Koob and Volkow, 2010).

Goal-directed behavior is, in part, controlled by dopamine (DA) release from the ventral tegmental area (VTA) into the nucleus accumbens (NAc) and the prefrontal cortex (PFC). This circuit is known as mesocorticolimbic system. Depending on its release site, DA codes for reward, anticipation, or motivation, and mediates associative learning or the activation of goal-directed behavior (Volkow et al., 2007). The reinforcing effects of addictive substances arise from their ability to stimulate DA release in mesolimbic areas, which substantially exceeds that caused by natural rewards, thereby creating strong incentive salience of drug cues (Hyman and Malenka, 2001). With repeated drug intake, epigenetic modifications alter gene expression profiles in mesocorticolimbic areas, leading to homeostatic adaptations. These include changes in synaptic morphology and dysregulation of dopamine receptors (Volkow et al., 2007; Robison and Nestler, 2011; Bidwell et al., 2019). Also, induction of the opioid peptide dynorphin inhibits DA release in the NAc *via* its action on κ-opioid receptors (Nestler, 2001). As a consequence of these alterations, the responsiveness of the reward system, and thus the motivation to pursue natural rewards, diminishes (Willuhn et al., 2010). Furthermore, in the later stages of addiction, mesocortical DA release initiates drug seeking (Kalivas and Volkow, 2005).

The PFC exerts top-down control over subcortical structures, like the striatum or the amygdala, and by that mediates higher-order cognitive functions, such as selection and coordination of complex behaviors as well as emotional regulation (Jackson and Moghaddam, 2001; Funahashi and Andreau, 2013). As addiction progresses, the ability of the PFC to choose behavioral options that favor long-term positive outcomes is compromised and immediate drug reward is preferred (Dalley et al., 2011). A structural correlate of this dysfunction is gray matter reduction in the PFC, which likely results from loss of dendritic complexity and spine density (Abernathy et al., 2010; DePoy et al., 2014; Mackey et al., 2018). Such atrophies correlate with the duration of drug abuse and impulsivity scores, implying disruption of executive function in the cortex as an underlying mechanism of escalating drug use (Qiu et al., 2013; Becker et al., 2015; Gröpper et al., 2016). Morphological alterations in the PFC change its neurotransmission, importantly leading to hyperactive efferents to the NAc. These corticoaccumbal projections become active upon mesocortical DA release and activate drug seeking (Kalivas and Volkow, 2005). A molecular hallmark of excessive corticoaccumbal transmission is significant down-regulation of metabotropic glutamate receptor subtype 2 (mGluR2) on cortical neurons (Meinhardt et al., 2013, 2021). mGluR2 serves as an inhibiting autoreceptor that modulates glutamatergic transmission (Kalivas et al., 2009). Cortical mGluR2 deficit was shown to be necessary and sufficient for impaired cognitive flexibility and elevated cue-induced drug seeking in ethanol-dependent rats (Meinhardt et al., 2021). Reduced mGlur2 expression was also found in human addicts and implied to play a role in other SUDs besides alcoholism (Jin et al., 2010; Meinhardt et al., 2013; Qian et al., 2019).

Besides that, the emotion and stress systems are crucially involved in the pathogenesis of SUDs (Koob and Schulkin, 2019). Regulation of affective states is achieved through prefrontal top-down control of the dorsal raphe nucleus (DRN) and the amygdala and is compromised in addiction (Vollenweider and Kometer, 2010; Lee et al., 2012; Wilcox et al., 2016). Amygdala and DRN control the activity of the hypothalamic–pituitary–adrenal (HPA) axis by modulating serotonin (5-hydroxytryptamine, 5-HT), corticotropin-releasing factor (CRF), and norepinephrine release in the hypothalamus (Feldman and Weidenfeld, 1998; Lowry, 2002). The HPA-axis mediates endocrine responses to stress and becomes increasingly sensitized during addiction, particularly during phases of abstinence (Koob and Schulkin, 2019). This seems to arise from excessive excitatory input from the extended amygdala, which in turn is attributable to a disruption of inhibitory control mechanisms within this structure (Kallupi et al., 2013; Stamatakis et al., 2014; Sharp, 2017). Consequently, the levels of stress-related neurotransmitters increase and negative affect becomes a driving force behind drug use (Koob, 2013). Stress-induced drug consumption is mediated by projections from the extended amygdala to the VTA and the NAc (Shaham et al., 2000; Kalivas and Volkow, 2005; Rinker et al., 2017). Structural and functional imaging studies support the notion that impaired prefrontal control over emotion and stress systems contributes to defective regulation of behavior (Li and Sinha, 2008; Qiu et al., 2013; Xiao et al., 2015). Additionally, chronic stress itself conduces to deleterious structural alterations and might exacerbate neuronal atrophy, and thus impairments in behavioral regulation, through reduction of dendritic complexity (Ansell et al., 2012; Lu et al., 2021).

To summarize, loss of control over drug consumption results from a series of adaptations in neurocircuits that regulate goal-directed behavior, executive function, and affective states. Abnormalities in all described circuits, resulting from genetic or environmental influences, have been suggested to elevate addiction vulnerability (Goldstein and Volkow, 2011; Shumay et al., 2012; Al’Absi et al., 2021).

## Effects of psychedelics – Prefrontal plasticity

Agonism at the 5-HT-2A receptor (5HT2AR) is thought to be the most relevant mechanism of serotonergic hallucinogens for eliciting psychoactive effects and lasting behavioral changes (Benko and Vrankova, 2020; DiVito and Leger, 2020; Pędzich et al., 2022). 5HT2ARs are abundantly expressed at dendrites of glutamatergic PFC pyramidal neurons in layer V, projecting to regions such as the amygdala, VTA, or NAc (Mocci et al., 2014; de Veen et al., 2017; Nichols and Hendricks, 2020). Thus, one common hypothesis regarding the anti-addictive properties of psychedelics focuses on induced neuroplasticity in the PFC leading to functional and structural alterations which may help to recover the phenotype (Ross, 2012; DiVito and Leger, 2020; Peters and Olson, 2021).

A variety of pathways and immediate early genes related to cell growth are upregulated by psychedelics, including brain-derived neurotrophic factor (BDNF) (Olson, 2022). BDNF has been repeatedly suggested to be the key effector in inducing neuroplasticity and thus lasting changes in cognition and behavior (Perkins et al., 2021; Knudsen, 2022; Olson, 2022). This is, for example, underlined by the fact that the anti-depressive effects of the dissociative ketamine, which displays therapeutic effects similar to psychedelics, are blocked by BDNF knockout in mice (Autry et al., 2011). Comparable studies with psychedelics are still missing (Olson, 2022). However, BDNF-driven plasticity seems to be the convergent mechanism of ketamine and serotonergic hallucinogens, hence it is not unlikely that BDNF is equally crucial for the lasting behavioral effects of classical hallucinogens (Aleksandrova and Phillips, 2021). The suggested process behind psychedelic-induced plasticity is initiated by 5HT2AR agonism, leading to the depolarization of prefrontal pyramidal neurons and locally increased glutamate release in the PFC (Muschamp et al., 2004). This drives activation of glutamatergic α-amino-3-hydroxy-5-methyl-4-isoxazole propionic acid (AMPA) receptors and subsequent BNDF secretion (Jourdi et al., 2009). BDNF binds to tropomyosin receptor kinase B (TrkB), resulting in activation of mechanistic target of rapamycin (mTOR), which in turn upregulates BDNF synthesis in dendrites, thereby creating a positive feedback loop that allows for prolonged periods of plasticity (Takei et al., 2004; Ly et al., 2021; Olson, 2022). Such periods are characterized by increases in neurito-, spino-and synaptogenesis, which might counteract neuronal atrophy occurring throughout addiction, and hence improve executive functioning and affective regulation (Ly et al., 2018; Aleksandrova and Phillips, 2021; Raval et al., 2021).

Activity on 5HT2AR-mGluR2 dimers may be crucially important for therapeutic effects of psychedelics (Benko and Vrankova, 2020; Meinhardt et al., 2021). As described above, mGluR2 deficit is an important hallmark of uncontrolled drug consumption, associated with impaired cognitive flexibility and elevated craving in ethanol-dependent rats. Psilocybin administration rescued this behavioral phenotype by restoring mGluR2 expression and presumably rebalancing aberrant corticoaccumbal glutamate transmission (Meinhardt et al., 2021). The persistence of these changes and their relevance in human patients need to be validated in human studies. In addition to corticoaccumbal modulation, prefrontal 5HT2AR stimulation *via* psychedelics is known to acutely increase activity of serotonergic neurons in the DRN and dopaminergic neurons in the VTA (Puig et al., 2003; Pehek et al., 2006). Long-lasting changes in these projections are yet to be identified but might contribute to regaining control over affective states and impulses, respectively.

Adaptation of 5HT2AR density in prefrontal areas could constitute another important effect of psychedelic action (Vollenweider and Kometer, 2010; Bogenschutz and Johnson, 2016). As elevated 5HT2AR availability in the PFC is associated with mood dysregulation and impulsivity, which are known features of SUDs, normalizing 5HT2AR density could alleviate symptoms of addiction (Frokjaer et al., 2008; Shelton et al., 2009; Rosell et al., 2010). Rapid induction of tolerance, known as tachyphylaxis, is a commonly observed phenomenon with psychedelics and is often accompanied by downregulation of 5HT2AR signaling in cortical areas (Buckholtz et al., 1988; Gresch et al., 2005; Raval et al., 2021). Although this presents regulation of 5HT2AR as a compelling mechanism, 5HT2AR availability returns to baseline a few days after psychedelic intervention in preclinical studies, which speaks against this effect as a mediator of lasting change (Buckholtz et al., 1990; Raval et al., 2021). PET studies in humans could not identify a reliable trend regarding neocortical 5HT2AR availability, although individual-specific relationships between 5HT2AR regulation and therapeutic outcomes were implied by Erritzoe et al. (2011) and Madsen et al. (2020). Further studies are necessary to evaluate the role of 5HT2AR modulation in therapeutic effects of psychedelics and explore alternative mechanisms besides downregulation, such as redistribution within the cell.

Various clinical findings support the idea of functional and structural plasticity in the PFC as a key mechanism of psychedelic therapy. For example, studies on regular users of the DMT-containing plant brew ayahuasca showed enhanced cognitive capabilities related to executive functioning as well as increased cortical thickness in the anterior cingulate cortex, a region that is crucial for affect regulation (Stevens et al., 2011; Bouso et al., 2012, 2015). Causality between the use of psychedelics and cognitive improvements mediated by effects in the cortex is further implied by increases in cognitive flexibility and altered metabolism in the anterior cingulate cortex following psilocybin intervention in depressive patients (Doss et al., 2021). Clinically controlled studies for long-term structural effects of psychedelics are lacking but could generate valuable insights, specifically with a focus on patients suffering from mood or addictive disorders. Higher capacity for emotional regulation, as well as lower levels of anxiety and depressive moods, were found in studies comparing frequent users of psychedelics to controls (Thiessen et al., 2018; Lafrance et al., 2021). In a clinical imaging study, psilocybin reduced reactivity of the amygdala to affective stimuli beyond acute drug effects. This was accompanied by increased activity of prefrontal regions known to control amygdala responses, which implies a potential improvement in top-down control over emotional reactions (Barrett et al., 2020).

## Effects of psychedelics – Direct effects on reward and stress systems

5HT2AR is also found in regions of reward and stress systems and directly contributes to regulation of goal-directed behavior and emotional states (de Veen et al., 2017; Nichols and Hendricks, 2020). Therefore, direct stimulation of these circuits might have therapeutic benefits besides top-down effects. One line of thought points toward effects of psychedelics on the mesolimbic DA system (Liester and Prickett, 2012; Ross, 2012; DiVito and Leger, 2020). Acutely, psychedelics elevate mesolimbic DA release, although not to a degree that renders them addictive (Vollenweider et al., 1999; Yan et al., 2000; Ross, 2012). Since persistent low-grade activity on dopamine 2 receptors (D2Rs) caused D2R upregulation in preclinical studies, it has been put forward that psychedelics could normalize D2R deficiency in the NAc by this effect (Boundy et al., 1995; Ross, 2012). Increases in D2R density caused by psychedelics were, indeed, identified in cell membranes from the mouse striatum. This effect was mediated by 5HT2AR, with which D2R forms heterodimers, and potentially caused by allosteric modulation of D2R signaling (Borroto-Escuela et al., 2014, 2021). As in the PFC, 5HT2AR availability in the striatum diminishes significantly after administration of psychedelics (Buckholtz et al., 1985). Since 5HT2AR positively regulates DA release in the mesolimbic pathway and repeated treatment with drugs of abuse seems to increase 5HT2AR sensitivity in the NAc, 5HT2AR downregulation might contribute to rebalancing of aberrant dopaminergic transmission (Pessia et al., 1994; Yan et al., 2000; Alex and Pehek, 2007). The persistence of changes in striatal DA release, D2R, and 5HT2AR density, as well as correlating behavioral alterations, requires further investigation. The same is true for changes in dynorphin levels. However, so far, one preclinical study implies that ayahuasca reduces the effects of ethanol on dynorphin activity (Almeida et al., 2022). Studies regarding psychedelic-induced 5HT2AR downregulation often focus on cortical areas, thus specific investigations in mesolimbic areas and their relation to therapeutic effects are lacking (Hall et al., 2000; Buchborn et al., 2015; Raval et al., 2021).

Furthermore, 5HT2AR is expressed in mood-regulating regions of the brain, such as amygdala or hypothalamus (Shi et al., 2008; Bombardi, 2014). Neuroimaging studies investigating acute effects of psychedelics in the amygdala revealed reduced activity during processing of fearful stimuli (Kraehenmann et al., 2015, 2016; Grimm et al., 2018). While such observations could be caused by top-down mechanisms, preclinical findings show that local stimulation of 5HT2AR in the amygdala is necessary and sufficient to suppress fear expression in rats, implying the presence of direct effects (Grimm et al., 2018; Barrett et al., 2020; Pędzich et al., 2022). Agonism at 5HT2ARs expressed at inhibitory interneurons in the amygdala could readjust their function and normalize hyperactive output to the stress system, like the hypothalamus, and thus re-balance the emotional state (Nichols and Hendricks, 2020). This effect could also decrease signaling to the VTA or the NAc and thereby stress-mediated drug seeking. Direct stimulation of 5HT2AR in the hypothalamus was shown to account for the neuroendocrine response to psychedelics, further supporting the notion that these substances display direct effects in stress-related regions (Hemrick-Luecke and Evans, 2002; Zhang et al., 2002). As use of psychedelics is associated with reduced levels of psychological distress in clinical and population studies, examinations of how this is reflected by long-term alterations in activity of the stress systems (e.g., HPA-axis) would be of interest (Hendricks et al., 2015; Johansen and Krebs, 2015; Doss et al., 2021).

## Conclusion

**Figure 1:**
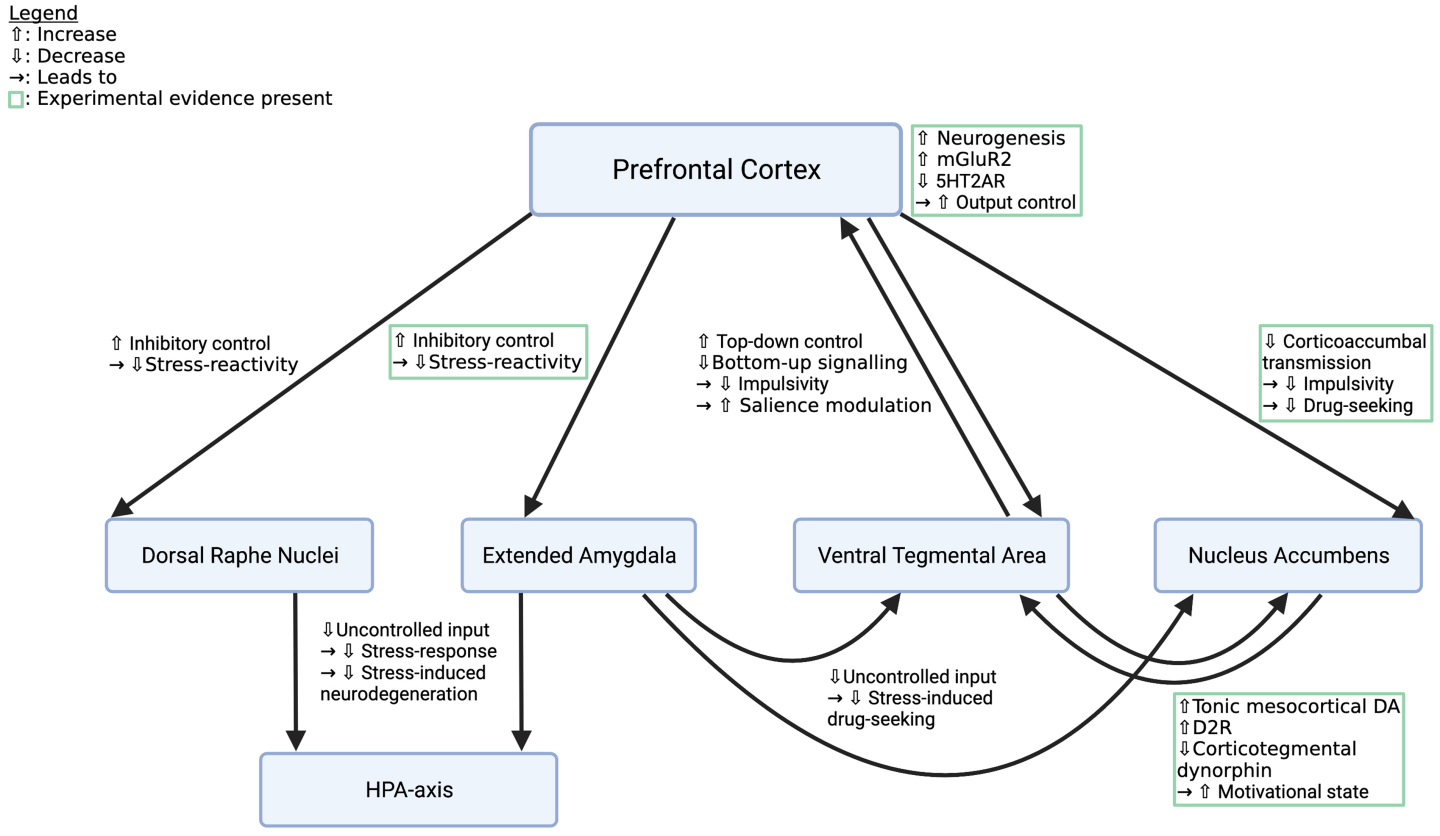
Effects of psychedelics on key pathways in the addicted brain. Depicted are crucial pathways that contribute to the behavioral and affective symptoms of SUDs and descriptions of how psychedelics supposedly alter their function to restore a healthy phenotype. Mechanisms listed in green boxes are backed up by experimental evidence, the other ones are deduced from knowledge about addiction circuitry and the effects of psychedelics. However, all pathways deserve closer examination. mGluR2, metabotropic glutamate receptor subtype 2; 5HT2AR, 5-hydroxy tryptamine 2a receptor; HPA-axis, hypothalamic–pituitary–adrenal axis. Created with BioRender.com.

Effects of psychedelics on addiction-related circuitry are diverse and include indirect as well as direct mechanisms in reward, stress, and emotion systems (see Table 1). Prefrontal plasticity supposedly re-establishes impaired top-down regulation of regions like the NAc, the VTA, DRN or the amygdala, which leads to increased control over emotions and impulses, thus reducing cue-and stress-induced drug intake and improving general mood (Vollenweider and Kometer, 2010; Bouso et al., 2015; Aday et al., 2020; see Figure 1). Specifically, rescue of mGluR2 expression was demonstrated to re-balance corticoaccumbal glutamate transmission and reduce craving (Meinhardt et al., 2021; see Figure 1). Direct effects in the limbic system might elevate DA-release and D2R-density, thereby normalizing the function of the reward system (Liester and Prickett, 2012; Ross, 2012; DiVito and Leger, 2020; see Figure 1). Acute effects in stress or emotion systems can partially be attributed to altered top-down regulation, however, local stimulation of the amygdala or the HPA-axis caused behavioral and neuroendocrine effects, respectively, as well (Zhang et al., 2002; Barrett et al., 2020; Pędzich et al., 2022). It is thus still unclear which proportion of the effects in subcortical structures are the consequence of top-down modifications and which part is caused *via* local action.

**Table 1:** Experimental evidence for psychedelic effects in key regions and pathways in the addicted brain.

Affected region/pathway	Species and psychedelic	Mode of exploration	Observed effects	References
PFC	Rat (DOI, LSD, DMT)	ELISA, ddPCR and morphology analyses after systemic application or in cell cultures	Increase in translated BDNF, spine and synapse density as well as dendritic branching	Ly et al. (2018, 2021)
PFC	Rat (LSD) Pig (psilocybin) Human (various psychedelics)	Radioligand binding assays after systemic application in animals; PET after systemic psilocybin application or lifetime psychedelic use in humans	Decreased 5HT2AR availability in preclinical studies; no or slight differences in 5HT2AR availability in clinical studies	Buckholtz et al. (1990), Gresch et al. (2005), Erritzoe et al. (2011), Madsen et al. (2020), and Raval et al. (2021)
PFC	Human (ayahuasca, psilocybin)	MRI of long-term ayahuasca users; fMRI/MRS in MDD patients treated with psilocybin	Increased ACC thickness in ayahuasca users; reduced ACC metabolism and altered connectivity after psilocybin	Bouso et al. (2015) and Doss et al. (2021)
PFC-NAc	Rat (psilocybin)	PCR and behavioral assays in ethanol-dependent rats after systemic application	Elevated mGluR2 expression and cognitive flexibility; reduced ethanol seeking	Meinhardt et al. (2021)
PFC-VTA	Rat (DOI)	Microdialysis after systemic application	Increased DA release in mesocortical pathway	Pehek et al. (2006)
PFC-Amyg	Human (psilocybin)	fMRI after systemic application	Reduced amygdala and increased prefrontal activity in response to aversive stimuli	Barrett et al. (2020)
PFC-DRN	Rat (DOI)	Electrophysiology and microdialysis after systemic and local application, respectively	Increased firing rate of PFC-DRN neurons after systemic and increased 5-HT release after local application	Puig et al. (2003)
VTA-NAc	Human (psilocybin) Rat (DOI)	PET and microdialysis after systemic application, respectively	Increased striatal D2R occupancy in humans; increased DA release in rats	Vollenweider et al. (1999) and Yan et al. (2000)
NAc	Human (LSD) Rat (DOI)	Radioligand binding assays in human cell cultures and rat striatum	Increased D2R density in cell culture and rat brain; decreased 5HT2AR binding in striatum of rat brain	Buckholtz et al. (1985) and Borroto-Escuela et al. (2014)
NAc-VTA	Mouse (ayahuasca)	Western blot after systemic application in ethanol-dependent animals	Decreased dynorphin concentration in withdrawal	Almeida et al. (2022)
Amygdala	Human (psilocybin) Mouse (DOI)	fMRI after systemic application in humans; behavioral assay after local application in mice	Decreased amygdala reactivity and altered connectivity in response to fearful stimuli in humans; suppressed fear expression in mice	Kraehenmann et al. (2015, 2016), Grimm et al. (2018) and Pędzich et al. (2022)
Hypothalamus	Rat (DOI)	Radioimmunoassay after systemic application	Increased corticosterone levels	Hemrick-Luecke and Evans (2002) and Zhang et al. (2002)

Summary of the experimental findings referenced in this paper. PFC, prefrontal cortex; DOI, 2,5-dimethoxy-4-iodoamphetamine; LSD, lysergic acid diethylamide; DMT, *N,N*-dimethyltryptamine; ELISA, enzyme-linked immunosorbent assay; (dd)PCR, (droplet digital) polymerase chain reaction; BDNF, brain-derived neurotrophic factor; PET, positron emission tomography; 5HT2AR, 5-hydroxy tryptamine 2a receptor; (f)MRI, (functional) magnetic resonance imaging; MRS, magnetic resonance spectroscopy; MDD, major depressive disorder; ACC, anterior cingulate cortex; NAc, nucleus accumbens; mGluR2, metabotropic glutamate receptor subtype 2; VTA, ventral tegmental area; DA, dopamine.

Studies employing local administration of psychedelics to or local blocking of 5HT2AR in important emotion-and reward-hubs in combination with animal models of addiction could shed light on the role of bottom-up mechanisms in subcortical structures. Furthermore, studies elucidating top-down effects on addiction circuitry are needed. These could include investigation of synaptic plasticity in corticolimbic or corticostriatal projections, examination of local transmitter release in response to different stimuli (e.g., fear-provoking or drug cues) pre versus post-psychedelics, and correlating structural changes with behavior. Most studies so far focus on acute or short-term effects of serotonergic hallucinogens and the field could benefit from (pre)clinical studies that systematically investigate long-term alterations in the key pathways outlined in this paper (see Figure 1). Despite the existing gaps, the current state of knowledge implies that psychedelics induce profound changes in cognition and emotional processing which are accompanied by circuit modifications that foster improvement of SUDs in general and challenge the efficacy of currently available addiction pharmacotherapy (Fuentes et al., 2020).

## Author contributions

MU collected the studies, wrote, and edited the manuscript, including creation of the figure and the table. MS and MM contributed to shaping the manuscript and editing it. All authors read and agreed upon the final manuscript.

## Funding

Financial support for this work was provided by Bundesministerium für Bildung und Forschung (BMBF) funded ERA-NET Psi-Alc (FKZ: 01EW1908), Deutsche Forschungsgemeinschaft (DFG, German Research Foundation) Project-ID: ME 5279/3-1 and the publication funding program ‘Open Access Publikationskosten’ by the University of Heidelberg and DFG.

## Conflict of interest

The authors declare that the research was conducted in the absence of any commercial or financial relationships that could be construed as a potential conflict of interest.

## Publisher’s note

All claims expressed in this article are solely those of the authors and do not necessarily represent those of their affiliated organizations, or those of the publisher, the editors and the reviewers. Any product that may be evaluated in this article, or claim that may be made by its manufacturer, is not guaranteed or endorsed by the publisher.
